# Cannabigerol–A useful agent restoring the muscular phospholipids milieu in obese and insulin-resistant Wistar rats?

**DOI:** 10.3389/fmolb.2024.1401558

**Published:** 2024-06-11

**Authors:** Patrycja Bielawiec, Sylwia Dziemitko, Karolina Konstantynowicz-Nowicka, Klaudia Sztolsztener, Adrian Chabowski, Ewa Harasim-Symbor

**Affiliations:** Department of Physiology, Medical University of Bialystok, Bialystok, Poland

**Keywords:** cannabigerol, insulin resistance, lipids, obesity, phospholipids, skeletal muscle

## Abstract

Numerous strategies have been proposed to minimize obesity-associated health effects, among which phytocannabinoids appear to be effective and safe compounds. In particular, cannabigerol (CBG) emerges as a potent modulator of the composition of membrane phospholipids (PLs), which plays a critical role in the development of insulin resistance. Therefore, here we consider the role of CBG treatment on the composition of PLs fraction with particular emphasis on phospholipid subclasses (e.g., phosphatidylcholine (PC), phosphatidylethanolamine (PE), phosphatidylserine (PS), and phosphatidylinositol (PI)) in the red gastrocnemius muscle of Wistar rats fed the standard or high-fat, high-sucrose (HFHS) diet. The intramuscular PLs content was determined by gas-liquid chromatography and based on the composition of individual FAs, we assessed the stearoyl-CoA desaturase 1 (SCD1) index as well as the activity of n-3 and n-6 polyunsaturated fatty acids (PUFAs) pathways. Expression of various proteins engaged in the inflammatory pathway, FAs elongation, and desaturation processes was measured using Western blotting. Our research has demonstrated the important association of obesity with alterations in the composition of muscular PLs, which was significantly improved by CBG supplementation, enriching the lipid pools in n-3 PUFAs and decreasing the content of arachidonic acid (AA), which in turn influenced the activity of PUFAs pathways in various PLs subclasses. CBG also inhibited the local inflammation development and profoundly reduced the SCD1 activity. Collectively, restoring the PLs homeostasis of the myocyte membrane by CBG indicates its new potential medical application in the treatment of obesity-related metabolic disorders.

## 1 Introduction

**SCHEME 1:**
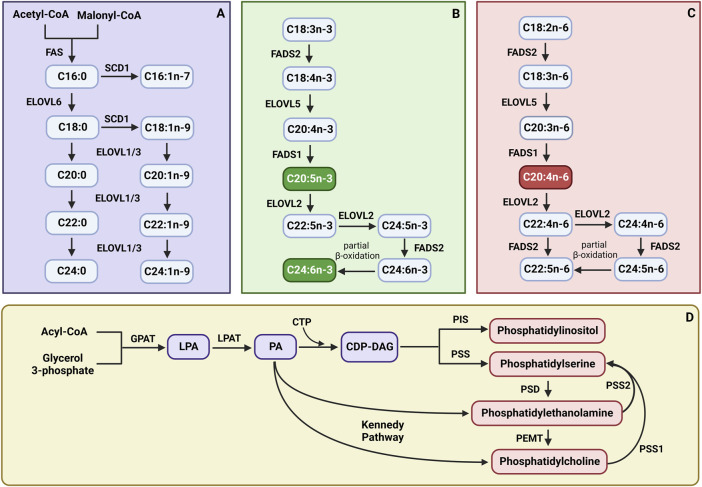
The synthesis pathways of **(A)** saturated fatty acids (SFAs) and monounsaturated fatty acids (MUFAs); **(B)** n-3 polyunsaturated fatty acids (PUFAs); **(C)** n-6 polyunsaturated fatty acids (PUFAs); **(D)** phospholipid subclasses. CTP - cytidine nucleoside triphosphate; CDP-DAG, cytidine diphosphate diacylglycerol; ELOVL1, fatty acid elongase 1; ELOVL2, fatty acid elongase 2; ELOVL3, fatty acid elongase 3; ELOVL5, fatty acid elongase 5; ELOVL6, fatty acid elongase 6; FADS1, fatty acid desaturase 1; FADS2, fatty acid desaturase 2; FAS, fatty acid synthase; GPAT, glycerol-3-phosphate acyltransferase; LPA, lysophosphatidic acid; LPAT, lysophosphatidyl acyltransferase; PA, phosphatidic acid; PEMT, phosphatidylethanolamine N-methyltransferase; PIS, phosphatidylinositol synthase; PSD, phosphatidylserine decarboxylase; PSS, phosphatidylserine synthase; PSS1, phosphatidylserine synthase 1; PSS2, phosphatidylserine synthase 2; SCD1, stearoyl-Coenzyme A desaturase. Created with BioRender.com.

Obesity is now considered one of the most serious medical burdens worldwide, gaining the name of a global epidemic. The issue has become a significant driver of the increase in metabolic comorbidities, including insulin resistance (IR), type 2 diabetes mellitus (T2DM), cardiovascular diseases (CVDs), and non-alcoholic fatty liver disease (NAFLD) (Boubertakh et al., 2022). Part of the metabolic derangements associated with obesity is largely due to the excessive accumulation of lipids not only in adipocytes but also in other metabolically active tissues, such as skeletal muscle (Morales et al., 2017). Especially, such lipidic entities as diacylglycerols (DAGs) and ceramides (CERs) have been negatively correlated with the action of insulin in muscle cells, which constitute a hallmark of glucose and fatty acids (FAs) metabolism disturbances (Morales et al., 2017). However, different lines of evidence have indicated that the dysregulation also concerned the phospholipids (PLs) fraction, causing changes in the content of various PLs subclasses, such as phosphatidylcholine (PC), phosphatidylethanolamine (PE), phosphatidylserine (PS), and phosphatidylinositol (PI) (Tiwari-Heckler et al., 2018). In addition, during obesity, there are also observed shifts in the FAs profile of PLs, influencing the ratio of saturated (SFAs), monounsaturated (MUFAs), and polyunsaturated fatty acids (PUFAs), which is essential for the functionality of this fraction (Tiwari-Heckler et al., 2018). The saturation/desaturation pattern of the FAs is determined by the acyl-coenzyme A (CoA) desaturases family, which introduces a cis-double bond in the acyl chain of long-chain fatty acids (LCFAs) (Guillou et al., 2010). Due to the specific position of the double bond introduction in the fatty acyl chain, desaturases can be divided into two distinct subgroups, namely, Δ9-desaturases, also known as stearoyl-CoA desaturases (SCDs), and fatty acid desaturases (FADS), including Δ5- (FADS1) and Δ6-desaturase (FADS2) (Miyazaki et al., 2008). SCD1 is a critical determinant of intracellular MUFAs/SFAs balance since it is the rate-limiting enzyme catalyzing MUFAs biosynthesis, mainly palmitoleic acid (C16:1n-7) and oleic acid (C18:1n-9), from preferential SFAs substrates, e.g., palmitic acid (C16:0) and stearic acid (C18:0), respectively (Liu et al., 2011). Accordingly, referring to the FADS1 and FADS2 enzymes, they participate in maintaining the PUFAs status, by activating the conversion of diet-derived essential FAs (linoleic acid (LA, 18:2n-6) and α-linolenic acid (ALA, 18:3n-3)) into the n-3 and n-6 series of PUFAs (Scheme 1) (Glaser et al., 2010). In the above processes, an important role is also played by elongases (elongation of very long-chain fatty acids, ELOVLs), the enzymes that extend fatty acyl-CoA by adding two carbon units (Jakobsson et al., 2006; Guillou et al., 2010). Evidence suggests that elongases show substrate specificity, which is related to the formed products and their metabolic roles (Guillou et al., 2010). Indeed, it has been established that the elongation reaction of SFAs and MUFAs can be achieved with ELOVL1,3,6 and 7, whereas ELOVL2 and 5 are selective for PUFAs utilization (Scheme 1) (Jakobsson et al., 2006). Consequently, obesity-related derangements in desaturation and elongation processes may result in PLs remodeling, which strongly affects membrane fluidity and activates several signaling pathways that might promote pro-inflammatory responses (Wu and Ballantyne, 2017). In particular, a disturbed membrane lipid homeostasis towards the n-6 series of PUFAs leads to the overproduction of 20-carbon PUFAs-derived eicosanoids, through the activation of distinct enzymes, such as cyclooxygenases (COXs) and lipoxygenases (LOXs), resulting in the arachidonic acid (AA, C20:4n-6) cascade hyperactivity (Wang et al., 2021). Therefore, in the course of obesity, restoring the phospholipid milieu seems crucial in preventing inflammation development and subsequent IR onset.

Over the last few decades, a growing body of evidence has shown that obesity is closely related to the overactivation of the endocannabinoid system (ECS), which constitutes the internal complex system of signaling lipid mediators (Di Marzo, 2008). In recent years, its definition has been extended to the endocannabinoidome (eCBome), as it has been established that the ECS consists not only of the canonical cannabinoid receptors (CB_1_ and CB_2_) and their endogenous ligands called endocannabinoids (mainly anandamide–AEA and 2-arachidonoylglycerol–2-AG), but also of additional molecular targets, including some members of the transient receptor potential (TRP) channel family (e.g., TRPA1), various orphan G protein-coupled receptors (e.g., GPR18 and GPR55), and nuclear transcription factor family of peroxisome proliferator-activated receptors (e.g., PPARα and PPARγ) (Veilleux et al., 2019). The above findings have encouraged scientists to search for non-intoxicating compounds of plant origin influencing the eCBome, which may have potential therapeutic applications in combating obesity and its related metabolic complications. Much recent attention has been given to phytocannabinoids, a bioactive compounds isolated from the ancient herb *Cannabis sativa* (Bielawiec et al., 2020). Of particular interest is cannabigerol (CBG), which does not exhibit psychomimetic activity, and several findings have indicated that its pharmacology is directed toward molecular targets distinct from those of the most studied cannabinoids CBD and Δ^9^-THC (Calapai et al., 2022). This multidirectional action of CBG determines its anti-inflammatory and antioxidant properties, which have been demonstrated in many comprehensive studies (Jastrząb et al., 2022). Moreover, the latest research also indicates that CBG can modulate insulin sensitivity of several tissues by affecting molecular targets, such as PPARα/γ, suggesting the potential therapeutic benefits of CBG use in such devastating diseases as obesity or metabolic syndrome (Nachnani et al., 2021; Bzdęga et al., 2023).

Until now, questions regarding the specific role of CBG in skeletal muscle remain unanswered. Therefore, this prompted us to investigate the potential therapeutic influence of a 2-week CBG application on the intramuscular content of PLs with particular emphasis on phospholipid subclasses (e.g., PC, PE, PS, and PI) in rats during the high-fat, high-sucrose (HFHS) diet regime. Furthermore, we also assessed the individual FAs composition, the total content of SFAs, MUFAs, and PUFAs, and the SCD1 index in the above-mentioned lipid pools. Additionally, to evaluate the ability of CBG to reduce local inflammation in the skeletal muscle of rats with IR induced by the HFHS diet, we examined the activity of n-3 and n-6 PUFAs pathways as well as the total expression of various proteins involved in the inflammatory process.

## 2 Materials and methods

### 2.1 Experimental model

The local Ethical Committee for Animal Testing in Olsztyn, Poland (No. 19/2022) approved all experimental protocols for animal care, handling, and experimentation. Additionally, all procedures were performed in accordance with relevant guidelines and regulations, including ARRIVE guidelines. Male Wistar rats were obtained from the Center for Experimental Medicine of the Medical University of Bialystok, Poland. Animals were maintained in standard cages under a controlled temperature of 22°C ± 2°C and were exposed to a 12/12 light-dark cycle, with unrestricted access to a selected rodent diet and either water or sucrose solution. The rats were acclimatized for 1 week prior to the random allotment to four separate groups (each of 10 individuals). The experimental groups were as follows: (Boubertakh et al., 2022): Control group received a basal rodent diet and water; (Morales et al., 2017); CBG group received a basal rodent diet and water, and CBG; (Tiwari-Heckler et al., 2018); HFHS group received a high-fat diet (HFD) and 20% sucrose solution; (Guillou et al., 2010); HFHS + CBG group received HFD, 20% sucrose solution, and CBG. Basal diet (Labofeed B, Animal Feed Manufacturer “Morawski”, Kcynia, Poland; containing 2.74 kcal/g distributed in 8 kcal% from fat, 67 kcal% from carbohydrates, and 25 kcal% from protein) and HFD (cat. no.: D12492, Research Diets Inc., New Brunswick, NJ, United States; containing 5.24 kcal/g distributed in 60 kcal% from fat, 20 kcal% from carbohydrates, and 20 kcal% from protein) were administered for 6 weeks. Starting from the 4^th^ week of the feeding course, for the two following weeks, rats were receiving synthetic CBG (once daily 30 mg/kg of body weight; purity ≥ 99%, cat. no.: TN1465, TargetMol, Boston, MA, United States) dissolved in sesame seed oil (1 mL/kg of body weight) by intragastrical route. For the same period of time, appropriate control groups were given the sesame seed oil vehicle (1 mL/kg body weight). The CBG dose was adjusted according to information available in the literature (El-Alfy et al., 2010; Moore et al., 2023). At the end of the sixth week, after a 12-h overnight fasting, the rats were anesthetized by intraperitoneal (i.p.) administration of pentobarbital (80 mg/kg of body weight). The body weight of each animal was monitored throughout the entire study and we reported that rats during the HFHS feeding course demonstrated significantly elevated body mass in comparison with the control rats, however, the implementation of CBG treatment markedly reduced their body weight compared to the HFHS group alone, as we showed in our recent paper (Bzdęga et al., 2023). Blood samples were collected into heparinized tubes from the inferior vena cava and then underwent centrifugation to obtain plasma. Muscle samples (red gastrocnemius muscle) were taken from animals, visible fatty tissue was mechanically removed and then, the obtained samples were immediately cryopreserved in liquid nitrogen (−196°C) using pre-cooled aluminum tongs and stored at a temperature of −80 °C for further analysis.

### 2.2 Determination of lipid content in the skeletal muscle tissue

The intramuscular content of total phospholipids fraction (Total PL), as well as individual PLs subclasses, i.e., PC, PE, PS, and PI, was examined by means of gas-liquid chromatography (GLC). In brief, the muscle samples (*n* = 10 for each group) were powdered, and lipids were extracted using the well-established Folch’s protocol (Folch et al., 1987) in a chloroform-methanol solution (in a volumetric ratio of 2:1) containing butylated hydroxytoluene as an antioxidant and heptadecanoic acid as an internal standard. Next, after overnight extraction the total PL as well as PC, PE, PS, and PI fractions were separated by thin-layer chromatography (TLC) on glass chromatographic plates covered with silica gel (Silica Plate 60, 0.25 mm; Merck, Darmstadt, Germany) using a resolving solution of chloroform/methanol/acetic acid (50:37.5:3.5, vol/vol/vol). The scrapped gel bands corresponding to the targeted fractions were eluted and subjected to transmethylation using a 14% boron trifluoride-methanol solution. Thereafter, the obtained samples were extracted in pentane solution followed by evaporation in a stream of nitrogen, and then finally samples were suspended in hexane. Based on the standard retention times, individual fatty acid methyl esters were identified and quantified using GLC (Hewlett-Packard 5890 Series II gas chromatograph, HP-INNOWax capillary column). The total PL fraction, as well as PC, PE, PS, and PI fractions in the red muscle samples, were assessed as the sum of the individual fatty acid species contents, which include: myristic acid–C14:0, palmitic acid–C16:0, palmitoleic acid–C16:1, stearic acid–C18:0, oleic acid–C18:1, linoleic acid–C18:2, arachidic acid–C20:0, linolenic acid–C18:3, behenic acid–C22:0, arachidonic acid–C20:4, lignoceric acid–C24:0, eicosapentaenoic acid–C20:5, nervonic acid–C24:1, and docosahexaenoic–C22:6. The values were expressed as nanomoles per Gram of wet muscle tissue. Additionally, based on the content of individual fatty acids in the abovementioned fractions we calculated the total intramuscular amount of SFAs (the sum of C14:0, C16:0, C18:0, C20:0, C22:0, and C24:0), MUFAs (the sum of C16:1, C18:1, and C24:1), n-3 PUFAs (the sum of C18:3, C20:5, and C22:6), and n-6 PUFAs (the sum of C18:2 and C20:4). Furthermore, we also calculated the SCD1 ratio as the content of C18:1n-9 divided by the C18:0 amount. Lastly, the activity of the n-3 PUFAs pathway was assessed as the sum of C20:5 and C22:6 contents divided by C18:3 content, and the activity of the n-6 PUFAs pathway was calculated as the content of C20:4 divided by the content of C18:2.

### 2.3 Western blotting

Western blotting technique was applied to detect the expression of selected proteins. Red gastrocnemius muscle homogenates were prepared using a RIPA buffer at 4 °C containing a mix of protease and phosphatase inhibitors (Roche Diagnostics GmbH, Mannheim, Germany). The protein concentration in each muscle sample was measured using the bicinchoninic acid method (BCA) with BSA as a standard. The homogenates were then reconstituted in Laemmli buffer (Bio-Rad, Hercules, CA, United States). Equal amounts of proteins (30 µg) were loaded on CriterionTM TGX Stain-Free precast gels (Bio-Rad, Hercules, CA, United States) and subsequently transferred to nitrocellulose or PVDF membranes in wet and semi-dry conditions, respectively. Tris-buffered saline with Tween-20 (TBST) and 5% non-fat dry milk or 5% BSA were used to block the membranes, which were then incubated overnight at 4°C with selected primary antibodies, the details of which are presented in Table 1. In the next step, bound antibodies were immunoblotted with the appropriate secondary antibody conjugated to horseradish peroxidase (HRP) (Cell Signaling Technology, Danvers, MA, United States), and, finally, the bands were visualized by chemiluminescence using the appropriate substrate (Clarity Western ECL Substrate; Bio-Rad, Hercules, CA, United States) and quantified densitometrically with Image Laboratory Software Version 6.0.1 (Bio-Rad, Hercules, CA, United States). The protein expression was assessed with stain-free gels and the total protein normalization method (Bio-Rad, Hercules, CA, United States). Each experimental group (*n* = 6) was expressed relatively (%) to the control.

**TABLE 1:** Primary antibodies used for the Western blotting method.

Target protein	Catalog number	Dilution	Vendor
5-LOX	sc-133085	1:500	Abcam, Cambridge, United Kingdom
12/15-LOX	sc-133085	1:500	Santa Cruz Biotechnology, Inc., Dallas, TX, United States of America
COL-1A1	sc-2931821	1:200	Santa Cruz Biotechnology, Inc., Dallas, TX, United States of America
COL-1A3	sc-514601	1:100	Santa Cruz Biotechnology, Inc., Dallas, TX, United States of America
COX-1	sc-19998	1:500	Santa Cruz Biotechnology, Inc., Dallas, TX, United States of America
COX-2	sc-166475	1:500	Santa Cruz Biotechnology, Inc., Dallas, TX, United States of America
cPLA2	sc-454	1:200	Santa Cruz Biotechnology, Inc., Dallas, TX, United States of America
ELOVL3	sc-54878	1:200	Santa Cruz Biotechnology, Inc., Dallas, TX, United States of America
ELOVL5	sc-398653	1:200	Santa Cruz Biotechnology, Inc., Dallas, TX, United States of America
ELOVL6	sc-385127	1:200	Santa Cruz Biotechnology, Inc., Dallas, TX, United States of America
FADS1	ab126706	1:500	Abcam, Cambridge, United Kingdom
FADS2	ab232898	1:500	Abcam, Cambridge, United Kingdom
MMP-2	sc-13595	1:500	Santa Cruz Biotechnology, Inc., Dallas, TX, United States of America
MMP-9	sc-393859	1:500	Santa Cruz Biotechnology, Inc., Dallas, TX, United States of America
NF-κB	cs-4764s	1:500	Cell Signaling Technology Inc., Danvers, MA, United States of America
Nrf2	sc-365949	1:500	Santa Cruz Biotechnology, Inc., Dallas, TX, United States of America
PPARγ	sc-7196	1:500	Santa Cruz Biotechnology, Inc., Dallas, TX, United States of America
SCD1	sc-14720	1:200	Santa Cruz Biotechnology, Inc., Dallas, TX, United States of America

### 2.4 Statistical analysis

The collected data are expressed as mean values ±SD or percentage of the control group. The analysis was conducted using GraphPad Prism version 8.2.1. for Windows (GraphPad Software, San Diego, CA, United States). The Shapiro-Wilk and Bartlett’s tests were used to evaluate the distribution of values and homogeneity of the variance. Two-way ANOVA followed by a *post hoc* test was used to determine statistical differences between groups. Any differences with *p*-value <0.05 were considered statistically significant.

## 3 Results

### 3.1 The impact of 2-week CBG administration on the intramuscular content of the total phospholipid fraction and various phospholipid subclasses: PC, PE, PS, and PI in the red gastrocnemius muscle

**FIGURE 1:**
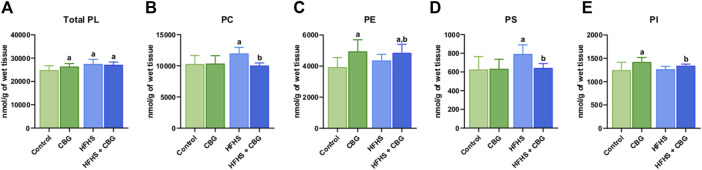
The content of total phospholipid fraction (Total PL) **(A)** and various phospholipid subclasses, i.e., phosphatidylcholine (PC) **(B)**, phosphatidylethanolamine (PE) **(C)**, phosphatidylserine (PS) **(D)**, and phosphatidylinositol (PI) **(E)** in the control group (rats fed a standard chow) and high-fat-high-sucrose diet (HFHS) group after 2-week cannabigerol (CBG) administration in the red gastrocnemius muscle. Values are expressed in nmol/g of wet tissue. The data are expressed as mean values ±SD and are based on ten independent determinations in each group (*n* = 10); ^a^p < 0.05 indicates a significant difference: the control group vs the examined group; ^b^p < 0.05 indicates a significant difference: HFHS group vs. HFHS + CBG group.

As presented in Figure 1, the Total PL content was significantly heightened in animals receiving HFHS feeding in relation to the control group (+10.6%, *p* < 0.05; Figure 1A). Simultaneously, 2-week CBG administration was found to increase the total PL level in both the group fed standard chow and the HFHS diet in comparison with the control rats (+5.1% and +9.4%, *p* < 0.05, respectively; Figure 1A). Additionally, CBG treatment meaningfully lowered the content of PC and PS (−16.2% and −18.9%, *p* < 0.05, vs the HFHS group, respectively; Figures 1B, D) that was raised by a HFHS diet (+16.4% and +26.5%, *p* < 0.05, vs the control group, respectively; Figures 1B, D). Interestingly, CBG elevated the level of PE and PI (+25.6% and +14.0%, *p* < 0.05, vs the control group, respectively) in standard diet-fed rats. In regard to HFHS, CBG administration also raised PI (+5.5%, *p* < 0.05, vs the HFHS group) and PE levels (+23.3% and +11.4%, *p* < 0.05, vs the control and HFHS groups, respectively; Figures 1C, E).

### 3.2 The impact of 2-week CBG administration on the intramuscular FAs composition in the total phospholipid fraction and various phospholipid subclasses: PC, PE, PS, and PI in the red gastrocnemius muscle

Our study revealed pronounced changes in the FAs composition of the total PL pool induced by HFHS feeding (Table 2). There was a substantial increase in the intramuscular content of C14:0, C16:0, C18:0, C18:1, C22:0, C20:4, and C22:6 in the total PL fraction, whereas we simultaneously observed a decrease in the C18:3 compound in comparison to the control group. Additionally, SFAs, MUFAs, n-3 PUFAs as well as n-6 PUFAs were raised (SFAs: +19.1%, MUFAs: +38.6%, n-3 PUFAs: 17.1%, and n-6 PUFAs: +7.7%; *p* < 0.05, vs the control group; Table 2), whereas the n-6/n-3 ratio was diminished in HFHS compared to the animals fed a standard chow (−10.7%, *p* < 0.05; Table 2). Administration of CBG led to substantial changes in C16:1, C18:0, C18:1, and C20:5 in comparison to rats fed either the standard or the HFHS diet. The changes were also found in C14:0, C16:0, C18:2, C18:3, C22:0, C20:4, and C22:6 compared to control conditions. Interestingly, SFAs and MUFAs levels were markedly diminished (−6.5% and −13.0%, *p* < 0.05, respectively; Table 2) after 2-week CBG treatment compared to the HFHS group; however, the content of SFAs, MUFAs, and n-3 PUFAs in the same experimental group was heightened (+11.3%, +20.6%, and +13.7%, *p* < 0.05, respectively; Table 2) in comparison to the control group. Concomitantly, the n-6/n-3 ratio was diminished (−12.8%, *p* < 0.05; Table 2) compared to rodents fed a basal diet. In standard feeding conditions, CBG was able to significantly influence only the content of the C18:1 FA component.

**TABLE 2:** Individual fatty acid composition of the total phospholipid fraction (Total PL) in the control group (rats fed a standard chow) and high-fat-high-sucrose diet (HFHS) group after 2-week cannabigerol (CBG) administration in the red gastrocnemius muscle. Values are expressed in nmol/g of wet tissue. The data are expressed as mean values ± SD and are based on ten independent determinations in each group (*n* = 10); ^a^p < 0.05 indicates a significant difference: the control group vs the examined group; ^b^p < 0.05 indicates a significant difference: HFHS group vs HFHS + CBG group. SFAs, saturated fatty acids; MUFAs, monounsaturated fatty acids; PUFAs, polyunsaturated fatty acids.

Total PL (nmol/g)	Control	CBG	HFHS	HFHS + CBG
C14:0	62.07 ± 4.99	68.12 ± 9.37	79.53 ± 8.37^a^	74.94 ± 8.75^a^
C16:0	5840.85 ± 331.95	6098.29 ± 284.87	6459.52 ± 401.89^a^	6285.54 ± 403.46^a^
C16:1	241.60 ± 46.79	238.58 ± 54.85	240.54 ± 49.20	188.06 ± 44.27^a,b^
C18:0	4575.96 ± 460.27	4909.45 ± 334.83	5778.62 ± 551.22^a^	5345.82 ± 217.25^a,b^
C18:1	994.18 ± 83.39	1105.03 ± 96.99^a^	1451.79 ± 111.88^a^	1338.29 ± 87.19^a,b^
C18:2	7234.08 ± 678.04	7617.12 ± 887.35	6850.54 ± 748.55	6395.54 ± 437.43^a^
C20:0	19.95 ± 2.96	20.53 ± 4.65	21.05 ± 4.77	21.76 ± 3.79
C18:3	39.40 ± 3.66	42.75 ± 12.73	30.93 ± 4.80^a^	30.76 ± 3.85^a^
C22:0	150.64 ± 16.22	147.07 ± 24.53	195.32 ± 29.75^a^	181.75 ± 31.86^a^
C20:4	3224.15 ± 257.62	3314.89 ± 175.42	4062.94 ± 319.43^a^	3992.33 ± 353.81^a^
C20:5	56.82 ± 10.05	56.17 ± 7.58	55.39 ± 10.79	45.60 ± 8.57^a,b^
C24:0	33.01 ± 6.86	31.71 ± 11.19	31.12 ± 8.18	38.08 ± 9.07
C24:1	36.16 ± 8.35	35.30 ± 9.56	31.59 ± 7.56	33.89 ± 3.40
C22:6	2351.33 ± 297.19	2441.42 ± 273.47	2780.53 ± 282.64^a^	2705.57 ± 299.61^a^
SFAs	10682.48 ± 766.42	11275.17 ± 548.26	12721.34 ± 807.86^a^	11986.97 ± 561.85^a,b^
MUFAs	1271.94 ± 124.62	1378.92 ± 136.47	1723.92 ± 146.83^a^	1560.24 ± 129.74^a,b^
n-3 PUFAs	2447.54 ± 305.74	2540.34 ± 277.05	2866.85 ± 286.19^a^	2781.93 ± 300.49^a^
n-6 PUFAs	10458.23 ± 817.64	10932.01 ± 917.17	11266.89 ± 756.26^a^	10301.86 ± 665.52^b^
n-6/n-3	4.31 ± 0.40	4.37 ± 0.74	3.82 ± 0.21^a^	3.76 ± 0.29^a^

In our experiment, a HFHS diet markedly elevated C18:0, C18:1, C22:0, C20:4, as well as C22:6, and diminished C20:0 and C18:3 content in comparison to the control group in PC fraction (Table 3). The amount of SFAs, MUFAs, n-3 PUFAs, and n-6 PUFAs increased (+11.0%, +31.7%, +59.6%, and +19.6%, *p* < 0.05, vs the control group, respectively; Table 3), and the n-6/n-3 ratio decreased after high-fat, high-sucrose treatment (−23.4%, *p* < 0.05; Table 3) compared to standard chow-fed rats. Chronic CBG treatment meaningfully changed the level of most of the examined components, namely,: C14:0, C16:0, C16:1, C18:0, C18:1, C18:2, C18:3, C22:0, C20:4, as well as C22:6 compared to HFHS group and C16:1, C18:1, C18:2, C18:3, C22:0, C20:4, C20:5, and C22:6 compared to control rats. The CBG was found to reduce the quantity of SFAs (−18.4%), MUFAs (−17.5%), n-3 PUFAs (−13.9%) as well as n-6 PUFAs (−16.4%) compared to the HFHS group (*p* < 0.05; Table 3). Moreover, the observed level of n-3 PUFAs was elevated (+37.4%, *p* < 0.05; Table 3) and the n-6/n-3 ratio was lessened (−24.1%, *p* < 0.05; Table 3) compared to subjects from the control group. Administration of CBG to rats on standard chow resulted in a greater amount of C20:5 FA compound in comparison to the standard chow-fed animals.

**TABLE 3:** Individual fatty acid composition of the phosphatidylcholine (PC) fraction in the control group (rats fed a standard chow) and high-fat-high-sucrose diet (HFHS) group after 2-week cannabigerol (CBG) administration in the red gastrocnemius muscle. Values are expressed in nmol/g of wet tissue. The data are expressed as mean values ± SD and are based on ten independent determinations in each group (*n* = 10); ^a^p < 0.05 indicates a significant difference: the control group vs the examined group; ^b^p < 0.05 indicates a significant difference: HFHS group vs HFHS + CBG group. SFAs, saturated fatty acids; MUFAs, monounsaturated fatty acids; PUFAs, polyunsaturated fatty acids.

PC (nmol/g)	Control	CBG	HFHS	HFHS + CBG
C14:0	33.17 ± 5.56	30.25 ± 5.71	36.06 ± 2.88	31.83 ± 4.08^b^
C16:0	4115.64 ± 512.44	3942.92 ± 521.79	4420.93 ± 409.50	3750.03 ± 244.06^b^
C16:1	149.71 ± 32.41	120.19 ± 49.90	146.07 ± 28.03	112.27 ± 39.15^a,b^
C18:0	1054.54 ± 178.96	1023.48 ± 147.07	1237.76 ± 185.24^a^	902.89 ± 162.19^b^
C18:1	622.57 ± 74.78	647.55 ± 90.63	870.70 ± 62.35^a^	726.12 ± 55.30^a,b^
C18:2	2529.55 ± 345.21	2597.32 ± 395.31	2359.89 ± 175.47	1985.15 ± 130.01^a,b^
C20:0	11.06 ± 2.55	11.76 ± 2.31	8.41 ± 2.76^a^	10.23 ± 1.42
C18:3	20.22 ± 2.27	19.88 ± 3.39	13.94 ± 1.63^a^	11.27 ± 1.76^a,b^
C22:0	66.22 ± 9.60	65.40 ± 13.66	101.42 ± 13.46^a^	86.93 ± 11.40^a,b^
C20:4	1250.42 ± 215.06	1386.38 ± 221.01	2088.11 ± 296.09^a^	1794.81 ± 140.27^a,b^
C20:5	22.43 ± 3.66	26.14 ± 3.66^a^	20.91 ± 2.57	18.61 ± 2.82^a^
C22:6	419.40 ± 108.59	487.42 ± 154.37	702.44 ± 96.16^a^	608.64 ± 76.24^a,b^
SFAs	5280.63 ± 690.42	5073.82 ± 633.36	5863.39 ± 484.45^a^	4781.91 ± 304.64^b^
MUFAs	772.28 ± 99.74	767.74 ± 116.85	1016.78 ± 76.03^a^	838.39 ± 88.53^b^
n-3 PUFAs	462.05 ± 113.50	532.72 ± 159.41	737.28 ± 98.20^a^	637.97 ± 77.16^a,b^
n-6 PUFAs	3779.96 ± 509.34	3983.70 ± 485.78	4520.10 ± 274.18^a^	3779.96 ± 127.79^b^
n-6/n-3	7.95 ± 1.12	7.53 ± 1.79	6.09 ± 0.59^a^	5.99 ± 0.64^a^

Regarding PE fraction (Table 4), the high-fat, high-sucrose treatment also significantly influenced individual FAs composition, as follows C14:0, C16:0, C16:1, C20:0, C22:0, C20:4, and C22:6 quantity in relation to the control (Table 4). The level of n-3 PUFAs heightened (+50.2%) and the n-6/n-3 ratio lessened (−26.1%) in comparison to rats receiving a basal diet (*p* < 0.05; Table 4). Chronic CBG administration to HFHS-fed animals resulted in an increase in C22:0, C20:4, and C22:6 and a decrease in C14:0, C16:1, and C20:0 content compared to the control rats. In the same experimental group, we also noticed that both n-3 and n-6 PUFAs amount raised (+69.7% and +20.3%, vs the control group, *p* < 0.05; Table 4) and the n-6/n-3 ratio dropped (−29.5%, vs the control group, *p* < 0.05; Table 4). Interestingly, the level of n-3 PUFAs increased (+13.0%, *p* < 0.05; Table 4) in animals fed HFHS diet and treated with CBG in comparison to the HFHS group alone. Meaningful changes in C14:0, C18:1, C18:2, C18:3, C22:0, C20:4 C22:6 as well as MUFAs, n-3 and n-6 PUFAs (MUFAs: +18.2%, n-3 PUFAs: +40.9%, and n-6 PUFAs: +30.6%, *p* < 0.05; Table 4) contents were also observed after chronic CBG administration to rats fed standard chow in comparison to the control group.

**TABLE 4:** Individual fatty acid composition of the phosphatidylethanolamine (PE) fraction in the control group (rats fed a standard chow) and high-fat-high-sucrose diet (HFHS) group after 2-week cannabigerol (CBG) administration in the red gastrocnemius muscle. Values are expressed in nmol/g of wet tissue. The data are expressed as mean values ± SD and are based on ten independent determinations in each group (*n* = 10); ^a^p < 0.05 indicates a significant difference: the control group vs the examined group; ^b^p < 0.05 indicates a significant difference: HFHS group vs HFHS + CBG group. SFAs, saturated fatty acids; MUFAs, monounsaturated fatty acids; PUFAs, polyunsaturated fatty acids.

PE (nmol/g)	Control	CBG	HFHS	HFHS + CBG
C14:0	8.73 ± 1.06	10.21 ± 1.97^a^	7.08 ± 1.88^a^	7.60 ± 0.97^a^
C16:0	479.06 ± 49.54	593.37 ± 193.58	563.65 ± 95.29^a^	542.10 ± 116.67
C16:1	22.34 ± 4.49	24.28 ± 3.92	18.40 ± 3.68^a^	16.73 ± 3.59^a^
C18:0	1464.19 ± 258.82	1594.32 ± 178.83	1329.00 ± 124.84	1411.98 ± 200.17
C18:1	165.18 ± 23.58	197.29 ± 27.40^a^	161.89 ± 21.30	175.93 ± 17.81
C18:2	477.82 ± 74.29	618.14 ± 108.27^a^	449.80 ± 55.90	465.07 ± 46.76
C20:0	10.10 ± 6.48	10.30 ± 4.66	4.96 ± 0.85^a^	4.60 ± 0.89^a^
C18:3	4.18 ± 0.98	6.18 ± 1.85^a^	4.77 ± 1.32	4.28 ± 0.76
C22:0	12.27 ± 1.80	16.88 ± 2.51^a^	15.98 ± 3.03^a^	16.69 ± 2.15^a^
C20:4	458.25 ± 66.41	604.56 ± 97.02^a^	599.47 ± 84.83^a^	660.92 ± 70.41^a^
C20:5	17.03 ± 3.15	20.57 ± 3.96	11.02 ± 1.96	11.18 ± 0.95
C22:6	819.30 ± 171.92	1157.66 ± 239.02^a^	1269.20 ± 172.52^a^	1434.50 ± 165.31^a^
SFAs	1975.70 ± 299.87	2319.56 ± 415.04	1920.67 ± 169.77	1982.96 ± 309.71
MUFAs	187.52 ± 26.07	221.57 ± 30.25^a^	180.29 ± 24.05	192.66 ± 18.65
n-3 PUFAs	840.51 ± 174.19	1184.42 ± 242.69^a^	1262.35 ± 168.59^a^	1426.02 ± 173.98^a,b^
n-6 PUFAs	936.06 ± 137.82	1222.69 ± 197.35^a^	1049.27 ± 132.19	1126.00 ± 101.71^a^
n-6/n-3	1.13 ± 0.12	1.06 ± 0.21	0.83 ± 0.07^a^	0.80 ± 0.09^a^

Individual FAs composition of PS fraction was also markedly changed after induction of HFHS diet to experimental animals (Table 5). The quantity of C16:0, C16:1, C18:1, C18:3, C22:0, C20:4, and C22:6 was higher compared to animals on standard diet. In addition, the level of MUFAs, n-3 and n-6 PUFAs raised (+20.9%, +68.5%, and +35.5%, *p* < 0.05; respectively; Table 5), whereas the n-6/n-3 ratio was diminished (−14.1%, *p* < 0.05; Table 5) in the abovementioned group compared to the standard chow-fed rats. CBG showed the ability to decrease C16:0, C18:0, C18:1, C18:2, C22:0, C20:4, and C22:6 raised by heightened FAs administration in the diet. Moreover, CBG introduction in the rats subjected to HFHS diet feeding elevated the content of C18:3 and C22:6 in the PS fraction when compared with the control group. Additionally, SFAs, MUFAs, n-3, and n-6 PUFAs quantity was lower after CBG treatment in HFHS-fed rats (−18.3%, −20.0%, −15.4%, and −28.2%, *p* < 0.05, respectively; Table 5) compared to HFHS group alone; however, the level of n-3 PUFAs was higher (+42.5% *p* < 0.05; Table 5) when compared with the controls. Nevertheless, the n-6/n-3 ratio was decreased when compared with both the control and HFHS groups (−26.1% and −14.0%, *p* < 0.05, respectively; Table 5). In addition, we also found that chronic CBG administration to rats on standard chow caused a significant increase in C16:1 and C18:3 FAs content in relation to standard diet-fed rats untreated with CBG.

**TABLE 5:** Individual fatty acid composition of the phosphatidylserine (PS) fraction in the control group (rats fed a standard chow) and high-fat-high-sucrose diet (HFHS) group after 2-week cannabigerol (CBG) administration in the red gastrocnemius muscle. Values are expressed in nmol/g of wet tissue. The data are expressed as mean values ± SD and are based on ten independent determinations in each group (*n* = 10); ^a^p < 0.05 indicates a significant difference: the control group vs the examined group; ^b^p < 0.05 indicates a significant difference: HFHS group vs. HFHS + CBG group. SFAs, saturated fatty acids, MUFAs, monounsaturated fatty acids, PUFAs, polyunsaturated fatty acids.

PS (nmol/g)	Control	CBG	HFHS	HFHS + CBG
C14:0	6.76 ± 0.74	6.78 ± 1.74	6.35 ± 0.76	6.87 ± 1.33
C16:0	64.86 ± 24.73	70.88 ± 17.48	86.83 ± 17.14^a^	68.06 ± 20.50^b^
C16:1	5.38 ± 1.39	6.45 ± 1.15^a^	6.72 ± 1.53^a^	6.32 ± 1.67
C18:0	341.73 ± 62.29	337.52 ± 51.41	372.38 ± 47.85	321.39 ± 18.87^b^
C18:1	37.22 ± 9.00	34.94 ± 4.97	44.77 ± 6.24^a^	35.91 ± 5.71^b^
C18:2	29.27 ± 12.67	26.45 ± 8.08	31.28 ± 5.98	21.38 ± 3.48^b^
C20:0	5.29 ± 1.29	5.86 ± 1.08	6.07 ± 1.09	4.80 ± 1.80
C18:3	3.41 ± 1.01	6.29 ± 1.80^a^	4.75 ± 1.58^a^	6.01 ± 2.65^a^
C22:0	12.54 ± 2.09	11.93 ± 2.04	14.21 ± 1.22^a^	11.24 ± 2.73^b^
C20:4	31.63 ± 10.18	30.63 ± 7.49	51.24 ± 10.84^a^	37.88 ± 3.85^b^
C20:5	8.81 ± 1.50	9.57 ± 1.92	9.81 ± 2.09	9.62 ± 1.85
C22:6	80.15 ± 16.31	86.69 ± 21.67	144.88 ± 32.07^a^	113.99 ± 10.19^a,b^
SFAs	431.18 ± 89.17	432.97 ± 63.85	491.78 ± 56.16	401.87 ± 14.49^b^
MUFAs	42.60 ± 9.90	41.39 ± 5.62	51.50 ± 6.10^a^	41.18 ± 5.57^b^
n-3 PUFAs	92.37 ± 18.33	102.54 ± 22.94	159.44 ± 29.97^a^	129.62 ± 9.53^a,b^
n-6 PUFAs	60.90 ± 22.68	57.08 ± 14.92	82.52 ± 15.10^a^	59.26 ± 6.62^b^
n-6/n-3	0.62 ± 0.12	0.56 ± 0.13	0.53 ± 0.06^a^	0.46 ± 0.05^a,b^

We also observed that the HFHS feeding influenced the individual FAs composition of PI fraction (Table 6). The intramuscular content of C16:0, C18:1, and C18:2 was decreased, while the level of C20:4, C20:5, C22:6, n-3, and n-6 PUFAs (n-3 PUFAs: +79.9%, n-6 PUFAs: +34.6% *p* < 0.05; Table 6) was increased compared to the control group. However, in the same experimental group of animals, we observed that the n-6/n-3 ratio markedly dropped (−25.3%, *p* < 0.05, vs the control group; Table 6). 2-week CBG treatment of rats on HFHS diet led to a substantial decrease in C16:0, C16:1, and C20:0 FAs content and an increase in C20:5 and total n-3 PUFAs quantity (n-3 PUFAs: +5.3%, *p* < 0.05; Table 6) in comparison to the HFHS group. Additionally, the level of C16:0, C18:1, C18:2, and n-6/n-3 ratio (n-6/n-3 ratio: −32.0%) significantly dropped, whereas the content of C20:4, C20:5, C22:6, n-3 and n-6 PUFAs (n-3 PUFAs: +89.5%, n-6 PUFAs: +27.9% *p* < 0.05; Table 6) pronouncedly heightened in the rats receiving HFHS and CBG treatment in relation to the rats receiving basal diet. Moreover, in the PI fraction experimental animals fed standard chow and treated with CBG expressed higher content of C16:0, C18:1, C20:0, C18:4, C20:4, C22:6 as well as SFAs, MUFAs, n-3 PUFAs, and n-6 PUFAs (SFAs: +13.2%, MUFAs: +12.4%, n-3 PUFAs: +27.4%, and n-6 PUFAs: +16.7%, *p* < 0.05; Table 6) in comparison to control animals.

**TABLE 6:** Individual fatty acid composition of the phosphatidylinositol (PI) fraction in the control group (rats fed a standard chow) and high-fat-high-sucrose diet (HFHS) group after 2-week cannabigerol (CBG) administration in the red gastrocnemius muscle. Values are expressed in nmol/g of wet tissue. The data are expressed as mean values ± SD and are based on ten independent determinations in each group (*n* = 10); ^a^p < 0.05 indicates a significant difference: the control group vs the examined group; ^b^p < 0.05 indicates a significant difference: HFHS group vs HFHS + CBG group. SFAs, saturated fatty acids; MUFAs, monounsaturated fatty acids; PUFAs, polyunsaturated fatty acids.

PI (nmol/g)	Control	CBG	HFHS	HFHS + CBG
C14:0	7.18 ± 1.11	7.38 ± 1.88	6.15 ± 1.26	6.62 ± 0.78
C16:0	78.46 ± 9.04	103.18 ± 12.13^a^	61.97 ± 5.24^a^	69.59 ± 8.75^a,b^
C16:1	7.12 ± 2.61	8.77 ± 1.25	8.57 ± 2.03	7.01 ± 1.15^b^
C18:0	750.74 ± 100.74	814.27 ± 61.04	722.94 ± 58.95	728.62 ± 27.79
C18:1	28.88 ± 3.20	31.69 ± 3.00^a^	24.97 ± 3.12^a^	26.04 ± 2.69^a^
C18:2	42.23 ± 8.87	47.67 ± 6.23	29.61 ± 8.96^a^	27.47 ± 3.40^a^
C20:0	3.87 ± 1.24	4.93 ± 0.75^a^	4.68 ± 0.95	3.56 ± 0.56^b^
C18:3	4.34 ± 2.56	8.16 ± 2.31^a^	4.85 ± 1.55	4.05 ± 1.70
C22:0	36.32 ± 5.87	38.02 ± 7.90	37.45 ± 4.88	34.09 ± 4.58
C20:4	267.58 ± 38.65	313.97 ± 37.37^a^	383.15 ± 35.44^a^	370.63 ± 10.16^a^
C20:5	10.25 ± 1.83	11.51 ± 2.30	25.41 ± 3.56^a^	31.32 ± 5.05^a,b^
C22:6	20.90 ± 3.03	25.54 ± 6.16^a^	31.23 ± 3.24^a^	31.89 ± 3.34^a^
SFAs	854.98 ± 94.93	967.78 ± 61.28^a^	833.19 ± 61.32	844.39 ± 34.82
MUFAs	35.99 ± 5.22	40.46 ± 4.06^a^	33.53 ± 4.28	33.04 ± 3.32
n-3 PUFAs	35.50 ± 7.08	45.22 ± 8.86^a^	60.99 ± 2.80^a^	67.26 ± 8.06^a,b^
n-6 PUFAs	309.80 ± 45.19	361.64 ± 42.21^a^	416.96 ± 44.28^a^	396.24 ± 11.61^a^
n-6/n-3	8.81 ± 0.72	8.14 ± 1.00	6.58 ± 0.44^a^	5.99 ± 0.70^a^

### 3.3 The impact of 2-week CBG administration on the SCD1 activity in the total phospholipid fraction and various phospholipid subclasses: PC, PE, PS, and PI in the red gastrocnemius muscle

**FIGURE 2:**
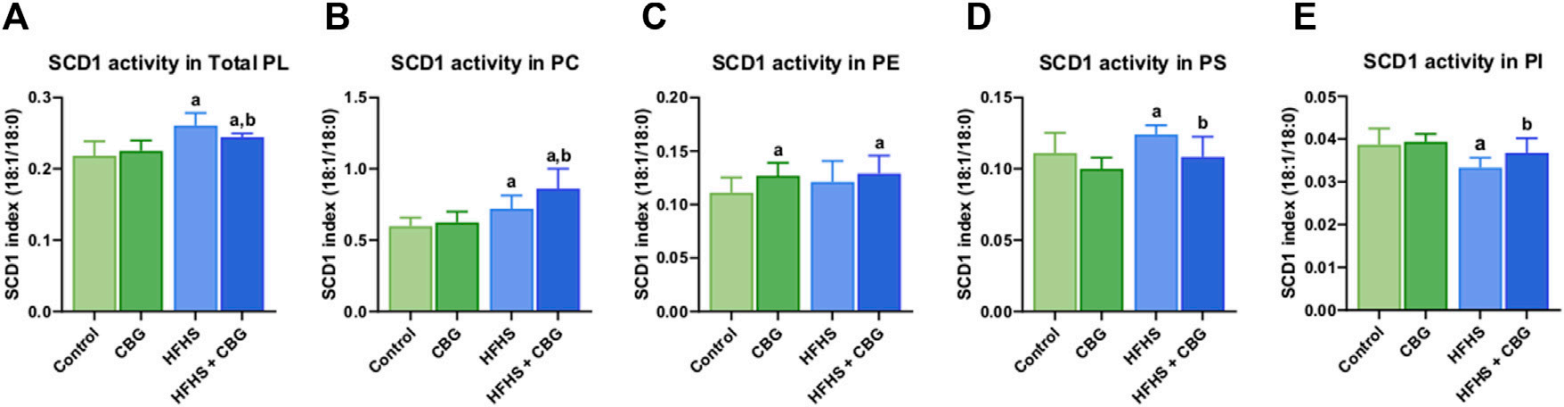
Intramuscular stearoyl-coenzyme A desaturase 1 (SCD1) activity in the total phospholipid fraction (Total PL) **(A)** and various phospholipid subclasses, i.e., phosphatidylcholine (PC) **(B)**, phosphatidylethanolamine (PE) **(C)**, phosphatidylserine (PS) **(D)**, and phosphatidylinositol (PI) **(E)** in the control group (rats fed a standard chow) and high-fat-high-sucrose diet (HFHS) group after 2-week cannabigerol (CBG) administration in the red gastrocnemius muscle. The SCD1 activity was calculated as the content of oleic acid (C18:1n-9) divided by the content of stearic acid (C18:0). The data are expressed as mean values ±SD and are based on ten independent determinations in each group (*n* = 10); ^a^p < 0.05 indicates a significant difference: the control group vs the examined group; ^b^p < 0.05 indicates a significant difference: HFHS group vs HFHS + CBG group.

Figure 2 shows that the activity of SCD1 was markedly altered in almost all of the examined phospholipid fractions, namely,: total PL, PC, PS, and PI, after the introduction of HFHS diet (+19.5%, +20.6%, +11.7%, and −13.9%, *p* < 0.05, vs the control group, respectively; Figure 2). 2-week CBG administration to rodents on HFHS diet reduced the SCD1 activity in total PL and PS (−6.4% and −12.6%, *p* < 0.05, respectively; Figures 2A, D) and heightened it in PC and PI pools (+19.4% and +10.5%, *p* < 0.05, respectively; Figures 2B, E) when compared to HFHS group alone. Additionally, the C18:1n-9/C18:0 ratio was increased in total PL fraction as well as PC, and PE (+11.9%, +44.1%, and +16.1%, *p* < 0.05, respectively; Figures 2A–C) in comparison with the control individuals. Notably, the SCD1 ratio was also considerably higher in animals on a standard diet after CBG introduction in PE fraction (+14.4%, *p* < 0.05, vs the control group; Figure 2C).

### 3.4 The impact of 2-week CBG administration on the total intramuscular expression of enzymes involved in FAs desaturation and elongation process in the total phospholipid fraction and various phospholipid subclasses: PC, PE, PS, and PI in the red gastrocnemius muscle

**FIGURE 3:**
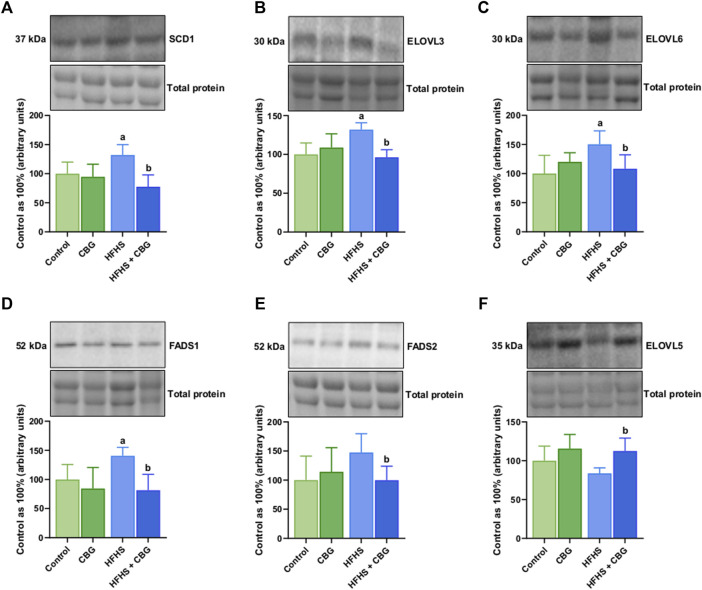
The total expression of proteins involved in fatty acid synthesis and metabolism: stearoyl-Coenzyme A desaturase 1 (SCD1) **(A)**, fatty acid desaturase 1 and 2 (FADS1 and FADS2) **(D,E)** as well as fatty acid elongase 3, 5 and 6 (ELOVL3, ELOVL5, and ELOVL6) **(B,F,C)** in the control group (rats fed a standard chow) and high-fat-high-sucrose diet (HFHS) group after 2-week cannabigerol (CBG) administration in the red gastrocnemius muscle. The total expressions of the abovementioned proteins are presented as percentage differences compared to the control group which was set as 100%. The data are expressed as mean values ±SD and are based on six independent determinations in each group (*n* = 6); ^a^p < 0.05 indicates a significant difference: the control group vs the examined group; ^b^p < 0.05 indicates a significant difference: HFHS group vs HFHS + CBG group.

As demonstrated in Figure 3, HFHS feeding was found to significantly increase the expression of SCD1, ELOVL3, ELOVL6, and FADS1 (+32.1%, +31.9%, +50.2%, and +40.6%, *p* < 0.05, respectively; Figures 3A–D) when compared to the control animals. Introduction of CBG to rats on an HFHS diet caused meaningful changes in the level of SCD1, ELOVL3, ELOVL6, FADS1, FADS2, and ELOVL5 (−41.3%, −27.0%, −27.8%, −41.9%, −34.3%, and +34.4%, *p* < 0.05, respectively; Figures 3A–F) in comparison to HFHS group alone.

### 3.5 The impact of 2-week CBG administration on the intramuscular activities of n-3 and n-6 polyunsaturated fatty acids (PUFAs) pathways in total phospholipid fraction, and in various phospholipid subclasses: PC, PE, PS, and PI in the red gastrocnemius muscle

**FIGURE 4:**
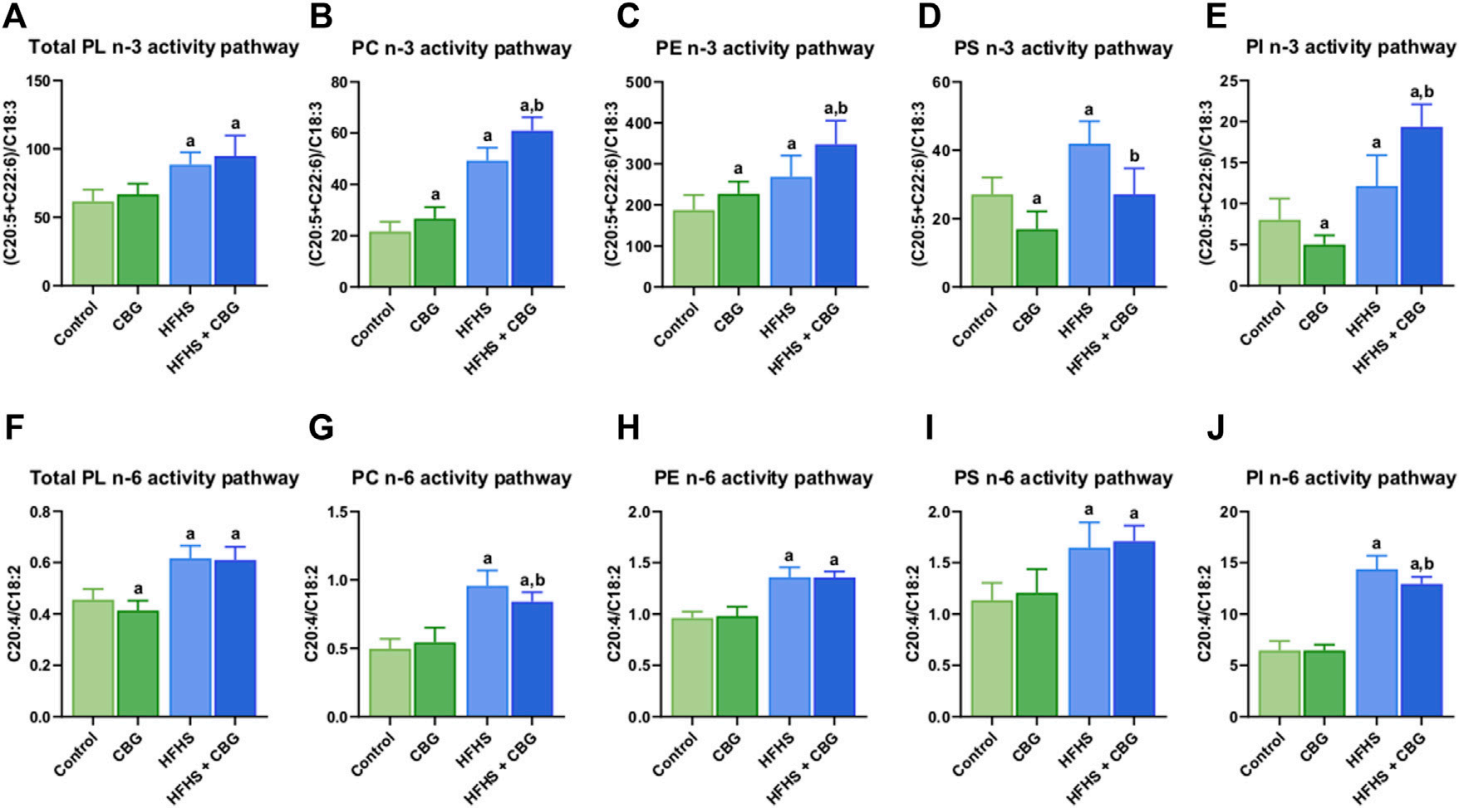
The activity of polyunsaturated fatty acids (PUFAs) n-3 and n-6 pathways in total phospholipid fraction (Total PL) **(A,F)** and various phospholipid subclasses, i.e., phosphatidylcholine (PC) **(B,G)**, phosphatidylethanolamine (PE) **(C,H)**, phosphatidylserine (PS) **(D,I)**, and phosphatidylinositol (PI) **(E,J)** in the control group (rats fed a standard chow) and high-fat-high-sucrose diet (HFHS) group after 2-week cannabigerol (CBG) administration in the red gastrocnemius muscle. The activity of the n-3 pathway was calculated as the sum of eicosapentaenoic acid (C20:5) and docosahexaenoic acid (C22:6) contents divided by linolenic acid (C18:3) content. The activity of the n-6 pathway was calculated as the content of arachidonic acid (C20:4) divided by the content of linoleic acid (C18:2). The data are expressed as mean values ±SD and are based on ten independent determinations in each group (*n* = 10); ^a^p < 0.05 indicates a significant difference: the control group vs the examined group; ^b^p < 0.05 indicates a significant difference: HFHS group vs. HFHS + CBG group.

**FIGURE 5:**
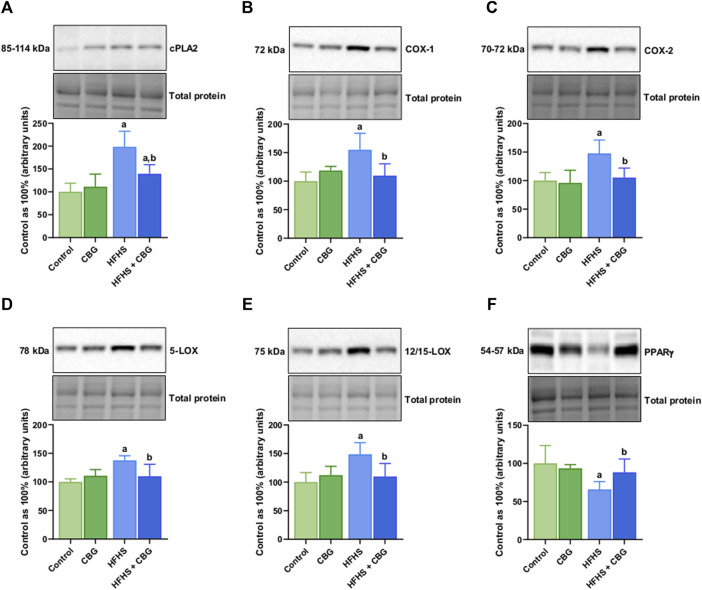
The total intramuscular expression of proteins involved in the eicosanoid synthesis pathway, i.e., cytosolic phospholipase A2 (cPLA2) **(A)**, cyclooxygenase 1 and 2 (COX-1 and COX-2) **(B,C)**, 5- and 12/15-lipoxygenase (5-LOXand 12/15-LOX) **(D,E)**, as well as peroxisome proliferator-activated receptor gamma (PPARγ) **(F)** in the control group (rats fed a standard chow) and high-fat-high-sucrose diet (HFHS) group after 2-week cannabigerol (CBG) administration in the red gastrocnemius muscle. The total expressions of the abovementioned proteins are presented as percentage differences compared to the control group which was set as 100%. The data are expressed as mean values ±SD and are based on six independent determinations in each group (n = 6); ^a^p < 0.05 indicates a significant difference: the control group vs the examined group; ^b^p < 0.05 indicates a significant difference: HFHS group vs HFHS + CBG group.

**FIGURE 6:**
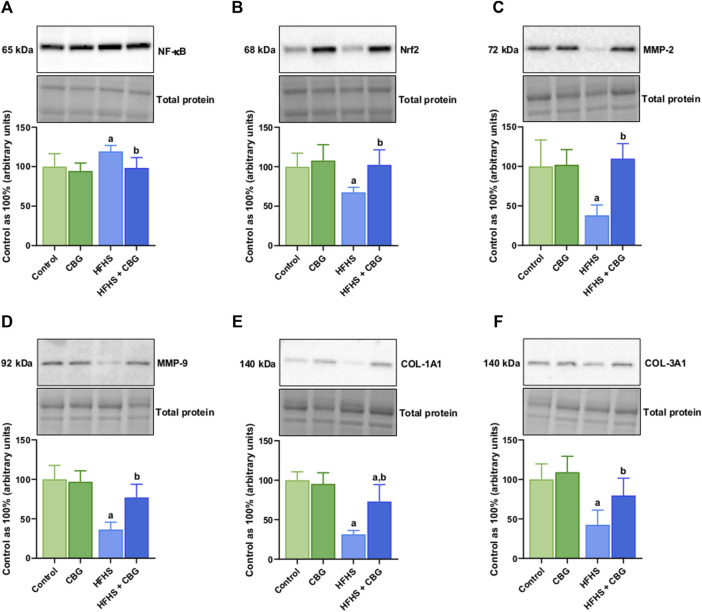
The total intramuscular expression of proteins involved in the inflammatory pathway, i.e., nuclear factor kappa B (NF-κB) **(A)**, nuclear factor erythroid 2-related factor 2 (Nrf2) **(B)**, matrix metalloproteinases 2 and 9 (MMP-2 and MMP-9) **(C,D)** as well as collagen type I, alpha 1 and collagen type III, alpha 1 (COL-1A1 and COL-3A1) **(E,F)** in the control group (rats fed a standard chow) and high-fat-high-sucrose diet (HFHS) group after 2-week cannabigerol (CBG) administration in the red gastrocnemius muscle. The total expressions of the abovementioned proteins are presented as percentage differences compared to the control group which was set as 100%. The data are expressed as mean values ±SD and are based on six independent determinations in each group (*n* = 6); ^a^p < 0.05 indicates a significant difference: the control group vs the examined group; ^b^p < 0.05 indicates a significant difference: HFHS group vs HFHS + CBG group.

In all examined phospholipid pools, as follows total PL, PC, PE, PS, and PI, a high-fat, high-sucrose feeding resulted in pronounced growth of activity of both PUFAs: n-3 (+43.9%, +127.3%, +43.2%, +54.9%, and +51.1%, *p* < 0.05, vs the control group, respectively, Figures 4A–E) and n-6 (+35.4%, +92.4%, +41.3%, +45.2%, and 122.5%, *p* < 0.05, vs the control group, respectively; Figures 4F–J) pathways in the skeletal muscle. In the group of HFHS diet-fed rats, the activity of the n-3 PUFAs pathway was intensified by CBG addition to animals in PC, PE, and PI fractions (+17.5%, +29.6%, and +59.4%, *p* < 0.05, vs the HFHS group, respectively; Figures 4B–E) but reduced in PS pool (−35.4%, *p* < 0.05, vs. the HFHS group). In turn, when compared with the control conditions, there was a significant increase in total PL, PC, PE, and PI fractions (+51.2%, +167.1%, +85.6%, and +140.8%, *p* < 0.05, respectively; Figures 4A–C, E). Furthermore, CBG treatment of standard diet-fed rodents led to higher n-3 PUFAs pathway activity in PC and PE (+23.3% and +21.1%, *p* < 0.05, vs the control group, respectively; Figures 4B, C) but lower in PS and PI fractions (−37.4% and 37.6%, *p* < 0.05, vs. the control group, respectively; Figures 4D, E). In regard to the n-6 PUFAs activity pathway, the introduction of CBG to HFHS diet-fed rats resulted in its diminished activity in PC and PI pools (−12.1% and −10.0%, *p* < 0.05, vs the HFHS group, respectively; Figures 4G, J). Similar to n-3 PUFAs, when compared to the control group, n-6 PUFAs were increased in total PL, PC, PE, PS, and PI pools (+34.1%, +69.2%, +41.2%, +50.8%, and +100.2%, *p* < 0.05, respectively; Figures 4F–J). A substantial difference in n-6 PUFAs activity after CBG administration to standard diet-fed animals was only observed in total PL fraction (−9.3%, *p* < 0.05, vs the control group; Figure 4F).

### 3.6 The impact of 2-week CBG administration on the total intramuscular expression of enzymes from the eicosanoid and prostanoid synthesis pathway and other proteins involved in the inflammatory process in the red gastrocnemius muscle

Figure 5 shows that the expression of all examined proteins involved in the inflammatory pathway, specifically cPLA2, COX-1, COX-2, 5-LOX, 12/15-LOX, and PPARγ was significantly influenced by HFHS feeding (+98.4%, +54.9%, +47.6%, +37.6%, +48.6%, and −34.2%, *p* < 0.05, respectively; Figure 5) compared to the standard diet-fed group. In rats receiving HFHS diet and simultaneously treated with CBG, there was a diminishment in the intramuscular expression of cPLA2, COX-1, COX-2, 5-LOX, and 12/15-LOX (−29.8%, −29.2%, −28.7%, −20.1%, and −26.2%, respectively; Figures 5A–E), and an increase in PPARγ (+34.3%, *p* < 0.05; Figure 5F) in relation to HFHS group alone. Additionally, in the same experimental group, the level of cPLA2 was raised in comparison to the control group (+39.4%, *p* < 0.05; Figure 5A). Moreover, in rats fed a HFHS diet we also observed a significantly affected the intramuscular expression of other proteins involved in the inflammatory pathways, such as NF-κB, Nrf2, MMP-2, MMP-9, COL-1A1, and COL-3A1 (+19.3%, −32.3%, −61.9%, −63.6%, −68.5%, and −57.4%, *p* < 0.05, vs the control group, respectively; Figure 6). Furthermore, as demonstrated in Figure 6, CBG was able to influence the expression of each examined protein, i.e., NF-κB, Nrf2, MMP-2, MMP-9, COL-1A1, and COL-3A1 in animals on HFHS chow (−17.5%, +51.5%, +188.3%, +111.4%, +132.1%, and +87.3%, *p* < 0.05, respectively; Figures 6A–F) compared to rodents receiving only HFHS diet. Lastly, the level of COL-1A1 (−26.9%, *p* < 0.05; Figure 6E) pronouncedly decreased in the abovementioned group in comparison to the control individuals.

## 4 Discussion

To the best of our knowledge, the following study is the first to propose new areas of interest for CBG’s medical use in obesity-related complications therapy, demonstrating that CBG treatment has a positive effect on the intramuscular phospholipids fraction by changing the content of individual PLs subclasses and modifying their FAs composition, which is associated with the attenuation of local inflammatory response in the skeletal muscle of insulin-resistant rats subjected to a high-fat, high-sucrose diet.

It is well-established, that obesity is associated with alterations in FAs metabolism, including partitioning between oxidation, storage, and changes in membrane structure, which in turn may affect the metabolic rate and initiate inflammatory processes (Zhang et al., 2010). Not surprisingly, in the present study, we noticed significant alterations in the composition of muscular PLs fraction under the FAs oversupply conditions. Specifically, the HFHS diet resulted in a marked elevation in the content of different PLs subclasses, e.g., PC and PS, which translates into the observed increase in the total intramuscular PLs pool. This is in accordance with a study of Funai et al., which demonstrated an increased PC concentration in the skeletal muscle of high-fat diet-fed mice (Funai et al., 2016). As they determined, it resulted from enhanced muscular expression of choline/ethanolamine phosphotransferase 1 (CEPT1), that is the step-limiting enzyme in the Kennedy synthesis pathway, through which PC as well as PE, are synthesized *de novo* (Funai et al., 2016; Tracey et al., 2018). In the same study, the authors also showed that skeletal muscle-specific CEPT1 knockout resulted in increased muscle sensitivity to insulin, which was linked to an altered composition of phospholipids in the sarcoplasmic reticulum and reduced sarco/endoplasmic reticulum Ca^2+^-ATPase (SERCA) activity (Funai et al., 2016). Interestingly, in the case of PE, we did not observe any significant changes under conditions of excess lipids, even though it shares the same Kennedy synthesis pathway as PC. This suggests increased interconversion of PE in favor of PC or PS since PS is only formed as a result of the *de novo*-produced PLs species remodeling by diverse deacylation/reacylation processes (Scheme 1) (Tracey et al., 2018). Importantly, the above-mentioned compositional changes in muscular PLs fraction during the increased consumption of FAs and sucrose were substantially improved by the 2-week CBG application. Our results show that the introduction of CBG in a group of insulin-resistant and obese Wistar rats significantly reduced the content of PC and PS. One of the possible explanations for the decrease in muscular PC content is the inhibitory effect of CBG on the activity of enzymes involved in the *de novo* PLs synthesis, e.g., CEPT1, which could result in improved muscular insulin sensitivity, however, this hypothesis requires further detailed research. In turn, in the case of PS, CBG probably intensifies its catabolism into PE, which corresponds to the elevated PE content in the group of animals subjected to the HFHS diet and CBG treatment. Additionally, we observed a considerable enhancement of intramuscular PE amount after CBG application not only in the group of HFHS diet-fed rats but also in rats fed the standard chow. It seems likely that this is another CBG’s cell protective mechanism as numerous studies have shown that PE serves as a key player in maintaining membrane integrity, cell cycle signaling as well as glucose and lipid metabolism pathways (Funai et al., 2016; Lee et al., 2018). This assumption is supported by a study conducted by Grapentine et al. in which they demonstrated that deficiency of PE synthesizing enzyme (CTP:phosphoethanolamine cytidylyltransferase - Pcyt2) in *Pcyt2* ^+/−^ mice results in disrupted PE milieu with subsequent deleterious metabolic effects including enhanced lipogenesis, greater intramuscular accumulation of DAGs and triacylglycerols (TAGs), impaired insulin signaling and mounting inflammatory state in skeletal muscle, which is linked to occurrence of obesity and IR (Grapentine et al., 2023). Moreover, the changes in muscle PC and PE content observed in our study are reflected in the PC:PE index, which was significantly reduced after the implementation of CBG treatment in both rats fed the basal and HFHS diets (data not shown). The above effects of CBG action should be emphasized since mounting evidence indicates that disruptions in the PC:PE tissue ratio caused by obesity are directly correlated with impaired glucose homeostasis, while correction of this ratio is associated with insulin sensitivity improvement (Fu et al., 2011; Bzdęga et al., 2023). This is in line with our previous publication, where we reported that the introduction of CBG to obese and insulin-resistant rats improved hepatic insulin sensitivity, which was reflected in increased Akt phosphorylation (in Ser and Thr phosphorylation sites), and hence, its activation, as well as in reduced phosphorylation of glycogen synthase kinase 3β (GSK-3β) in Tyr 216, resulting in decreased glycogen deposition (Bzdęga et al., 2023). Another important finding from our study is the discovery that 2-week CBG administration significantly increased intramyocellular amounts of PI after feeding rats both standard chow and HFHS diet. It is well established that PI is an essential component of the cell membrane that functions as a lipid anchor of numerous membrane proteins (Derkaczew et al., 2023). Furthermore, it also constitutes a precursor for many derivatives, such as phosphatidylinositol-3,4,5-trisphosphate (PIP_3_), that activates downstream signaling proteins, the most notable of which is the protein kinase B (PKB/Akt), which plays a pivotal role in the insulin signal transduction pathway (Derkaczew et al., 2023; Khezri et al., 2023). Therefore, taking into account the above-mentioned effects caused by the CBG treatment, it seems reasonable to conclude that this phytocannabinoid may prevent the development of IR in the skeletal muscle under the increased dietary FAs bioavailability conditions through improving the cellular phospholipids milieu.

Additionally, at the same time, we observed that in our study not only the intramuscular amounts of individual PLs subclasses changed but also the fatty-acyl chain composition differed significantly in these fractions. This seems to be extremely important since the results obtained in the studies conducted on both experimental animals and humans indicate that the FAs composition of the PLs fraction in the cell lipid bilayer is related to membrane fluidity as well as to insulin responsiveness of the skeletal muscle (Kriketos et al., 1996; Clore et al., 1998). However, most of the studies were carried out in the total membrane phospholipids, therefore we decided to evaluate the composition of individual FAs considering distinct PLs species. Our experimental model showed that under the conditions of the HFHS diet feeding, the content of SFAs, MUFAs, as well as n-3 and n-6 PUFAs in the PC pool increased substantially. Noteworthy, the largest increment concerned the content of n-6 PUFAs, which resulted from the significant enrichment of this fraction in arachidonic acid (AA; C20:4n-6). The large majority of studies describe the biological effects of AA as pro-inflammatory, demonstrating that it serves as a precursor of intermediates (termed eicosanoids) whose action orchestrates many detrimental processes involved in the pathogenesis of a wide range of metabolic disorders, including IR (Wang et al., 2021). The above statement corresponds with the research conducted by Koeberle et al. (Koeberle et al., 2013), which showed that supplementation of NIH 3T3 murine fibroblasts plasma membranes with AA-rich phosphatidylcholine species (20:4-PC) impaired downstream insulin-mediated signaling apparently by inhibiting the interaction of Akt with its PIP_3_ binding sites. Concomitantly, in the same study researchers showed that phosphatidylcholines enriched in SFAs and MUFAs did not affect the Akt signaling, suggesting that the ability of PC species to suppress the Akt pathway decreases with the unsaturation index of the forming FAs (Koeberle et al., 2013). Moreover, we should also bear in mind that AA constitutes a biosynthetic precursor of endocannabinoids, of which the best-studied are AEA and 2-AG (Di Marzo et al., 2008). These AA-derived metabolites are endogenous ligands of CB_1_ and CB_2_ receptors, and according to the current state of knowledge, overactivation of the CB_1_ receptor is closely related to the induction of the inflammatory response and occurrence of obesity (Silvestri and Di Marzo, 2013; Bielawiec et al., 2020). Additionally, our research also showed that the oversupply of FAs in the diet caused similar changes in the composition of the PS fraction as in PC, including a significant enhancement in the proportions of MUFAs, n-3, and n-6 ​​PUFAs. Despite a significant elevation in the AA content of the PS fraction, the most noticeable change concerned the amount of n-3 PUFAs, which resulted from a drastic increase in the content of docosahexaenoic acid (DHA; C22:6n-3), that has been linked to broad effects in promoting metabolic health (Hishikawa et al., 2017). One of the possible explanations for the high DHA content in the PS fraction is the significant preference for DHA-rich substrates by PS synthesizing enzymes, e.g., phosphatidylserine synthase 2 (PSS2) (Kimura and Kim, 2013). Meanwhile, both in the case of the PC and PS subclasses, 2-week CBG supplementation in animals during the HFHS feeding period resulted in a significant decrease in the content of all classes of FAs, as follows SFAs, MUFAs, and PUFAs, however, the largest diminishment among these fractions concerned the amount of AA, which confirms the anti-inflammatory properties of CBG. In contrast, the alternations in the PE and PI fractions observed in our experiment are completely different from those regarding the PC and PS subclasses. Specifically, our research has shown that either in the PE or PI species, the content of individual FAs in the group of HFHS-fed rats changed only slightly in relation to rats receiving a basal diet. In turn, chronic administration of CBG to rats during both a standard and HFHS feeding course resulted in prominent fatty-acyl chain modifications, which indicates an interesting phenomenon regarding the increased incorporation of PUFAs from the n-3 pathway into these fractions. The most probable explanation seems to be the activation of desaturation and elongation processes by CBG treatment, leading to the shift of the long-chain PUFAs equilibrium in favor of n-3, well-known as anti-inflammatory precursors. Thus, the above effects of CBG can be considered beneficial since it enhances the anti-inflammatory and antioxidant potential of PE and PI subclasses, which are abundantly localized on the inner leaflet of the cell membranes (Patel et al., 2017). Collectively, the present observations strongly suggest that the lipid bilayer phospholipids remodeling, including complex interconversions of PLs species and alternations in their FAs composition, is closely associated with myocyte insulin sensitivity. Therefore, restoring the lipid homeostasis of the myocyte membrane by CBG in conditions of the overabundance of FAs and carbohydrates in the diet seems to be extremely important and indicates a new potential medical use of CBG in the treatment of obesity-related metabolic disorders.

In accordance with the above findings is the enhanced muscular FADS1 and FADS2 expression in the group of animals fed the HFHS diet, which was subsequently reduced by following CBG treatment. FADS1 and FADS2 are the crucial step-limiting enzymes involved in the PUFAs metabolism, in particular, responsible for the multistep synthesis of AA as well as eicosapentaenoic acid (EPA; C20:5n-3) and DHA from the diet-derived essential FAs: LA and ALA, respectively (Scheme 1) (Glaser et al., 2010). Downstream conversions of FADS family products result in the formation of various lipid signaling mediators that participate in both the initiation and alleviation of inflammatory processes, which might modify whole body metabolic rate (Miyazaki et al., 2008). Furthermore, recent studies have indicated a positive interrelationship between enhanced FADS1 activity and obesity state (Powell et al., 2016; Yashiro et al., 2016). Consistently, Yashiro’s research group demonstrated that the use of a selective FADS1 inhibitor prevented obesity development in HFD-fed mice (Yashiro et al., 2016), whereas, another study showed that FADS1 gene knockout (*fads* ^−/−^) mice exhibited improved glycemic parameters and resistance against obesity induction under HFD-feeding conditions (Powell et al., 2016). Similarly, evidence accumulating during the last decades has shown that obesity, type 2 diabetes mellitus (T2DM), and distinct inflammatory diseases are also correlated with elevated FADS2 activity (Warensjö et al., 2006; Arshad et al., 2019). Wang et al. reported that male C57BL/6 J FADS2 knockout mice (*fads2* ^−/−^) were characterized by lower body weight, reduced adipocyte size, and enhanced lipolysis markers in comparison with wildtype mice regardless of the feeding regime (diet enriched with Lard or Flax) (Wang et al., 2023). Thus, data from our experiment support the hypothesis whereby upregulated FADS1 and FADS2 expression is associated with the occurrence of obesity and IR (insulin sensitivity results were demonstrated in our recently published paper (Bzdęga et al., 2023)). Therefore, we can assume that the observed reduction in intramuscular FADS1 and FADS2 expression is in line with the diminished AA concentration following the administration of CBG in obese rats points out the high therapeutic potential of CBG as a new class of drug useful in combating obesity-related complications. Intriguingly, we should also note that the most pronounced changes caused by CBG administration across all examined PLs fractions concern AA and DHA with no significant changes in EPA content. Hence, it seems likely that CBG promotes the process of fatty-acyl chain elongation, leading to the formation of very-long-chain PUFAs from 18- and 20-carbon substrates. Indeed, in the present study, we revealed that implementation of CBG in rats fed a high-fat, high-sucrose diet substantially elevated the intramuscular expression of ELOVL5 responsible for PUFAs chain elongation, which reflects the above-mentioned changes. This gains significant importance as recent research has shown that ELOVL5 knockout (*Elovl5* ^−/−^) mice developed hepatic steatosis via overactivation of sterol regulatory element-binding protein 1c (SREBP-1c) and its target genes (e.g., acetyl-CoA carboxylase–ACC and fatty acid synthase–FAS) involved in intracellular FAs synthesis (Moon et al., 2009). The underlying mechanism can be explained most likely due to a deficiency of endogenous AA and DHA, which are recognized to inhibit the activity of SREBP-1c, forming a negative feedback loop (Moon et al., 2009). Our results also demonstrated that the muscular expression of ELOVL3 and ELOVL6 in rats fed the HFHS diet was significantly upregulated compared to the control groups. These elongases preferentially utilize SFAs and MUFAs as substrates in the two-carbon units addition cycle (Guillou et al., 2010), specifically, ELOVL6 is involved in the formation of C18 long-chain SFAs and MUFAs, while ELOVL3 is responsible for C20 - C24 FAs production (Matsuzaka et al., 2007; Zach-Avec et al., 2010). In recent years, interest in the role of these enzymes has increased significantly, as a growing body of evidence has pointed to their direct connection with obesity, where they demonstrate intensified activity patterns. In accordance, the mice lacking ELOVL3 (*Elovl3* ^*−/−*^) developed by Zach-Avec and coworkers (Zach-Avec et al., 2010) were resistant to the induction of obesity by an HFD, which was associated with a reduced FAs uptake and inhibited *de novo* synthesis. In a similar manner, research on ELOVL6 identified that gene deletion in mice (*Elovl6* ^*−/−*^) ensured resistance against diet-induced IR, although the mice were characterized by obesity and hepatic steatosis (Matsuzaka et al., 2007). Noteworthy, the herein study has shown that treatment of obese and insulin-resistant rats with CBG caused a pronounced decrease in either ELOVL3 or ELOVL6 intramuscular expression. Additionally, when analyzing FAs metabolic pathways, we should not forget about another important enzyme, SCD1. It is responsible for catalyzing the MUFAs formation (mainly palmitoeleic acid (C16:1n-7) and oleic acid (C18:1n-9)) and appears to be a pivotal orchestrator of a wide range of metabolic processes including lipid synthesis and oxidation (Dobrzyn and Ntambi, 2004). Due to the multifaceted roles of MUFAs, alternations in SCD1 activity are expected to impact a variety of metabolic pathways, including those involved in insulin signal transduction and obesity (Sampath and Ntambi, 2011). Indeed, recently it has been proven that SCD1 deficient mice (*Scd1* ^*−/−*^) exhibit enhanced energy expenditure, improved insulin sensitivity of skeletal muscle, and high resistance to diet-induced weight gain, which collectively highlight the close link between SCD1 overactivity and obesity occurrence (Dobrzyn et al., 2005; Miyazaki et al., 2007). Thus, targeted SCD1 inhibition has the potential to protect against multiple aspects of obesity-associated metabolic derangements. Given the variety of beneficial effects of CBG action, our findings revealed that it additionally possesses the capability to significantly reduce the total intramuscular SCD1 expression in the HFHS diet-fed rats, which corresponds with the inhibition of the SCD1 activity in the total PLs and PS fraction in the same experimental group. One possible mechanism for diminished SCD1 activity after CBG implementation is the ability of CBG to effectively modulate endocannabinoid signaling since the latest research has shown a functional connection between the eCBome and the lipogenesis process (Liu et al., 2013). Over the past decade, increased CB_1_ receptor activation has been attributed to the promotion of lipogenic enzyme expression, such as SCD1 (Liu et al., 2013). Furthermore, it has been shown that products of SCD1, endogenous MUFAs downregulates the fatty acid amide hydrolase (FAAH) activity, resulting in impaired AEA degradation and subsequent CB_1_ overactivation, which constitutes a positive feedback loop (Liu et al., 2013). Therefore, we can assume that CBG interrupts this vicious cycle that occurs during obesity since CBG has been demonstrated to be a partial agonist of the CB_1_ receptor with low affinity and is considered as a strong competitive inhibitor of AEA (Nachnani et al., 2021). However, in the case of PC, PE, and PI species, we observed an increase in SCD1 activity index compared to animals receiving only the high-fat, high-sucrose diet. This reverse effect of CBG action in PC, PE, and PI subclasses is puzzling, and more experiments are needed to elucidate the exact mechanism. Moreover, it is also worth adding that in our previous research we showed results, that are consistent with the herein data and demonstrate that another phytocannabinoid, namely, cannabidiol (CBD), substantially decreased the ELOVL3 and ELOVL6 expression, as well as reduced the SCD1 activity ratio in different lipid pools in the skeletal muscle of rats with HFD-induced obesity (Bielawiec et al., 2023). Thus, this collectively suggests that phytocannabinoids are emerging as compounds that may represent a novel therapeutic approach for the treatment of obesity and accompanying IR through the modification of FAs-metabolizing enzymes activity involved in elongation and desaturation processes.

It has been generally accepted that the overabundance of FAs and carbohydrates in the diet is closely related to the occurrence of obesity, which is accompanied by inflammatory modifications resulting from the cell membrane PLs remodeling (Wu and Ballantyne, 2017). In the present study, analogously to the increased AA content in all examined PLs fractions during the course of obesity, we also reported a markedly elevated n-6 PUFAs activity pathway. Intriguingly, 2-week CBG administration in rats under the lipid overload conditions also promotes the upregulation of n-6 PUFAs activity in all PLs species in comparison with the control subjects, although compared to the rats fed HFHS diet alone, we noted profoundly decreased n-6 activity pathway only in the case of the PC and PI subclasses. Presumably, the possible explanatory mechanism could be an increase in the content of γ-linoleic acid (GLA; C18:3n-6), which serves as a precursor for AA formation. This notion is supported by the study of Vodolazska et al., where they demonstrated that a diet enriched with hemp seed oil caused a marked elevation in the GLA concentration, whose interconversion to AA may intensify the activity of the n-6 PUFAs pathway (Vodolazska and Lauridsen, 2020). Moreover, in our study, significant modifications of PUFAs activity also concerned the anti-inflammatory n-3 series. In particular, HFHS diet-fed rats (untreated and treated with CBG) have shown a substantial rise in intramuscular n-3 activity pathway in all PLs fractions except for PS. However, CBG application elevated the activity of n-3 PUFAs in the PC, PE, and PI classes to a greater extent compared to obese rats, indicating increased incorporation of FAs from the anti-inflammatory n-3 pathway. Thus, we strongly believe that CBG markedly improved the anti-inflammatory and anti-oxidative potential of membrane PLs by modifying the composition and activity of PUFAs, which is also reflected in alternations in the expression of enzymes involved in inflammatory pathways that we observed in our research. In accordance, during the high-fat, high-sucrose feeding course, we found significantly elevated intramuscular expression of cytosolic phospholipase A_2_ (cPLA2), an enzyme that plays a key role in PLs hydrolysis to AA (Leslie, 2015). Increased cPLA2 activity along with a relative excess of AA in the lipid bilayer may lead to its continuous release from membrane deposits and make it available for the generation of eicosanoids with the participation of enzymes from the COX or LOX family (Leslie, 2015). Not surprisingly, in the herein study, we noted that rats with HFHS diet-induced obesity have shown profoundly enhanced enzyme expression of COX-1, COX-2, 5-LOX, and 12/15-LOX, which are engaged in the production of various cytokines with high pro-inflammatory effects attributed to the development of local and systemic inflammatory response. This suggests the occurrence of inflammation in the skeletal muscle, which may be partially linked to the onset of diet-induced IR. It is worth emphasizing that, CBG implementation in the same group of animals substantially inhibits all AA-metabolizing enzymes, such as cPLA_2_, COX-1, COX-2, 5-LOX, and 12/15-LOX, which is in line with the study conducted by Ruhaak et al., demonstrating the inhibitory activity of CBG against both COX-1 and COX-2 enzymes with high effectiveness (Ruhaak et al., 2011). Presumably, one of the mechanisms that could account for the immunomodulatory properties of CBG is its ability to bind to the CB_2_ receptor and PPARγ (Jastrząb et al., 2022). Specifically, Giacoppo et al. in their study suggest that CBG through the CB_2_ receptor activation led to an increase in the cellular level of NF-κB inhibitor - IκB-α and, consequently, a decrease in the NF-κB activity (Giacoppo et al., 2017). Another possibility is the potent stimulation of PPARγ by CBG since it has been proven that upregulation of this transcription factor induces termination of the NF-κB signaling pathway, which leads to the suppression of genes involved in the initiation of the inflammatory response, including COX-2 (Hou et al., 2012). In line with the above hypothesis, our data showed significantly enhanced muscular PPARγ expression in HFHS diet-fed rats after CBG application in comparison with the untreated obese rats, which in turn corresponds to a significant reduction in the total NF-κB and COX-2 expression in the same group of experimental animals. In light of these findings, CBG may hold great promise for the treatment of chronic low-grade inflammatory states potentially via modulation of the activity of enzymes participating in the AA cascade, which most likely results in reduced generation of lipid pro-inflammatory mediators. Simultaneously, our immunoblotting analyses also revealed that lipid overabundance conditions substantially reduced the level of nuclear factor erythroid 2-related factor 2 (Nrf2), which is the central regulator of cellular resistance to oxidative stress (Ma, 2013). Importantly, 2-week CBG administration restored the Nrf2 activity in rats subjected to HFHS feeding, indicating the beneficial antioxidant properties of CBG, which, in addition to its anti-inflammatory activity, protects cells against obesity-induced damage. Moreover, we also have found that CBG inhibits the fibrotic muscular remodeling during the obesity state, which was revealed by a significant upregulation of secreted matrix metalloproteinases 2 and 9 (MMP-2 and MMP-9) as well as collagen type 1, and type 3 (COL-1A1 and COL-3A1) expression, which contributes to the regulation of extracellular matrix turnover and probably may increase the regenerative capacity of the skeletal muscles. Collectively, CBG favorably impacts the composition and metabolism of muscle PLs, which is associated with the inhibition of the AA-derived bioactive metabolites formation accounting for the beneficial effects of CBG therapy. Thus, our experimental study is the first step toward understanding the modulatory action of CBG on membrane PLs, however, further research should be introduced to determine the exact mechanism of CBG due to the considerable complexity of the above processes.

## 5 Conclusion

Taking the above into account, our results indicate that high-fat, high-sucrose diet-induced obesity significantly affects the dynamics of muscular PLs by changing the content of individual phospholipid subclasses and the composition of forming FAs via modification of the fatty-acyl chain, which is related to the activation of various signaling pathways that can promote pro-inflammatory responses. In addition, we provided new insight into the properties of CBG, demonstrating that it acts as a regulator of PLs metabolism, which is associated with attenuating the local inflammatory response in skeletal muscle. All of these findings revealed the promising potential of CBG therapy, indicating its possible therapeutic usefulness in the treatment of obesity-related metabolic disorders.

## Data Availability

The raw data supporting the conclusion of this article will be made available by the authors, without undue reservation.

## Abbreviations

5-LOX, 5 lipoxygenase; 12/15-LOX, 12/15 lipoxygenase; COL-1A1, collagen, type I, alpha 1; COL-3A1, collagen, type III, alpha 1; COX-1, cyclooxygenase 1; COX-2, cyclooxygenase 2; cPLA2, cytosolic phospholipase A2; ELOVL3, fatty acid elongase 3; ELOVL5, fatty acid elongase 5; ELOVL6, fatty acid elongase 6; FADS1, fatty acid desaturase 1; FADS2, fatty acid desaturase 2; MMP-2, matrix metalloproteinases 2; MMP-9, matrix metalloproteinases 9; NF-κB, nuclear factor kappa B; Nrf2, nuclear factor erythroid 2-related factor 2; PPARγ, peroxisome proliferator-activated receptor gamma; SCD1, stearoyl-Coenzyme A desaturase.
